# Gamma Knife radiosurgery to four or more brain metastases in patients without prior intracranial radiation or surgery

**DOI:** 10.1002/cam4.206

**Published:** 2014-02-10

**Authors:** Eric Ojerholm, John Y K Lee, James Kolker, Robert Lustig, Jay F Dorsey, Michelle Alonso-Basanta

**Affiliations:** 1 Department of Radiation Oncology, University of PennsylvaniaPhiladelphia, Pennsylvania; 2 Department of Neurosurgery, University of PennsylvaniaPhiladelphia, Pennsylvania

**Keywords:** Gamma Knife radiosurgery, radiation therapy neoplasm metastases, stereotactic radiosurgery, survival, tumor volume

## Abstract

Data on stereotactic radiosurgery (SRS) for four or more metastases are limited. Existing studies are confounded by significant proportions of patients receiving prior whole-brain radiation therapy (WBRT) or concurrent WBRT with SRS. Furthermore, published results disagree about the impact of tumor volume on overall survival. A retrospective review identified 38 patients without prior intracranial radiation or surgery who received Gamma Knife (GK) as sole treatment to ≥4 brain metastases in a single session. Twenty-eight cases with follow-up imaging were analyzed for intracranial progression. Prognostic factors were examined by univariate (log-rank test) and multivariate (Cox proportional hazards model) analyses. Common primary tumors were non-small cell lung (45%), melanoma (37%), and breast (8%). Cases were recursive partitioning analysis class II (94%) or III (6%). Patients harbored a median five tumors (range 4–12) with median total tumor volume of 1.2 cc. A median dose of 21 Gy was prescribed to the 50% isodose line. Patients survived a median 6.7 months from GK. Local treatment failure occurred in one case (4%) and distant failure in 22 (79%). On multivariate analysis, total tumor volume ≥3 cc was significantly associated with distant failure and worsened overall survival (*P* = 0.042 and 0.040). Fourteen patients (37%) underwent salvage WBRT at a median 10.3 months from GK and seven patients received repeat GK. GK as sole initial treatment for four or more simultaneous metastases spares some patients WBRT and delays it for others. Increased total tumor volume (≥3 cc) is significantly associated with worsened overall survival.

## Introduction

Brain metastases are a frequent occurrence in oncology patients. Optimal management is controversial and may include surgery, whole-brain radiation therapy (WBRT), and stereotactic radiosurgery (SRS). Prospective, randomized trials evaluating the role of SRS have been almost exclusively limited to patients with one to three brain metastases 1–6. Data on patients with four or more lesions are limited, and WBRT is currently standard of care in such cases 7,8. Concern about the neurocognitive impact of WBRT 3, however, has prompted investigators to examine SRS for patients with ≥4 lesions 9–18. These studies are complicated by significant proportions of patients treated with prior WBRT or concurrent WBRT with SRS. Published results furthermore disagree about prognostic factors for overall survival, in particular the impact of total tumor volume 9–12. In this study, we review our experience using Gamma Knife (GK) as the sole initial therapy for four or more simultaneous brain metastases in patients with no prior history of intracranial radiation or surgery. We examine overall survival, patterns of intracranial progression, and need for salvage therapies in this population, and we explore prognostic factors associated with these outcomes.

## Methods

### Data collection

We retrospectively reviewed the charts of 435 patients who underwent SRS using GK for brain metastases between 2006 and 2010 at Pennsylvania Hospital, Philadelphia, U.S.A. This study was approved by the University of Pennsylvania Institutional Review Board. We identified 38 patients who received GK as sole therapy to four or more metastases in a single session and had no prior history of intracranial radiation or surgery. Patient and disease characteristics were retrieved from the electronic medical record. Dates of death were confirmed with the Social Security Death Index or local newspaper obituaries. Extracranial disease was retrospectively classified as stable, progressive, newly diagnosed, or none. Patients were considered to have no extracranial disease if they received definitive treatment to the primary tumor and showed no disease on last pre-GK imaging. Patients were classified as newly diagnosed if brain metastases were discovered within 1 month of primary tumor diagnosis or if only a single recent set of pre-GK extracranial imaging was available. In cases without explicit documented performance status, we retrospectively assigned a value using history and physical exam notes from the date of treatment. We examined two volume variables: tumor volume and treatment volume. Both were calculated retrospectively using the GK planning software. Tumor volume for a single metastasis was defined as the volume of enhancing brain tissue correlating with that lesion on the T1-weighted postcontrast GK planning magnetic resonance imaging (MRI). This value was calculated by summing the contoured volume of the enhancing lesion. Total tumor volume for any patient denotes the sum of the individual tumor volumes for all metastases in that patient. Treatment volume for a single metastasis was defined as the volume of brain tissue receiving at least the prescribed marginal dose for that target lesion. Total treatment volume for any patient denotes the sum of the individual treatment volumes for all metastases in that patient.

### Radiosurgical technique

Radiosurgery was performed using a Model 4-C GK (Elekta, Inc, Stockholm, Sweden) with Gamma Plan software. A Leksell stereotactic headframe was applied using local anesthesia with conscious sedation. High-resolution 3D T1 images were acquired with gadolinium contrast. Flair axial MR sequences were used at 3-mm slices to image any associated edema. As detailed contours of the enhancing tumor volume were not available for all metastases and in order to avoid interplanner contour variability, all tumor volumes were contoured and calculated retrospectively by a single GK planner (E. O.) as part of this study. The plan was executed and patients were recovered in the GK suite and the frames removed as routine. Steroids were only administered at the individual decision of the treating physician. Antiepileptics were not routinely administered before or after the procedure.

### Follow-up evaluation

Patients were generally followed with clinic visits and MR imaging at 1–3 months posttreatment and then every 3 months thereafter.

### Data analysis

Overall survival from GK to death was calculated by the Kaplan–Meier method. All 38 cases were included in the analysis, with living patients censored at last clinical encounter. Patterns of intracranial failure were evaluated in the 28 patients (78%) with follow-up imaging. Of the remaining 10 cases, eight patients died and two cases were lost to follow-up prior to any post-GK imaging. Local failure was defined as interval radiologic growth of a previously treated lesion, while distant failure was defined as development of new parenchymal metastases away from treated sites or development of leptomeningeal disease. For both local and distant failure analyses, cases without intracranial progression were censored at the date of last brain MRI. For local failure analysis, patients who had first failed distantly and undergone salvage WBRT were censored at the date of last brain MRI prior to WBRT. Salvage therapies included WBRT, repeat GK, Cyberknife, and surgery. All 38 patients were included in the analysis of salvage therapies, with cases censored at date of death or, if lost to follow-up, at the date of last clinic visit. Associations between patient characteristics (age, gender) or disease characteristics (primary tumor type, extracranial disease status, number of metastases, location of metastases, time from primary tumor diagnosis to development of brain metastases, total tumor volume, total treatment volume) and the outcomes of overall survival and distant failure were explored. Recursive partitioning analysis (RPA) class was not included in this analysis due to the small number of patients with designations other than class II.

### Statistics

Statistical analysis was performed using STATA 12 (StataCorp, College Station, TX, USA). Univariate analysis was computed using the log-rank test. We examined continuous variables at a variety of cutoff points. Factors with *P* < 0.30 on univariate analysis were included in the multivariate analysis, which was calculated using the Cox proportional hazards model. Hazard ratios (HR) and 95% confidence intervals (CI) were noted, and statistical significance was reached if *P* < 0.05 on the multivariate analysis.

## Results

From 2006 to 2010, 38 patients without prior history of intracranial radiation or surgery received GK as sole initial treatment to four or more simultaneous metastases. Patient and disease characteristics are shown in Table 1. Eighteen men (47%) and 20 women (53%) underwent GK at a median age of 66 years. The primary tumor was non-small cell lung in 17 cases (45%), melanoma in 14 (37%), breast in three (8%), and a single case each of bladder, ovarian, renal cell, and mesothelioma. Extracranial disease was classified as progressive in 14 patients (37%), stable in five (13%), newly diagnosed in 17 (45%), and none in two patients (5%). RPA class was identified for 35 cases (92%), yielding zero class I patients, 33 class II patients, and two class III patients. Five of these patients were categorized retrospectively. Three patients remained unclassified for RPA because their performance status could not be determined by history or physical exam notes from the time of treatment. The median interval between primary tumor diagnosis and discovery of brain metastases was 4.2 months. Prior to GK planning scan on the day of treatment, only 10 patients (26%) were known to have four or more brain metastases. The remaining patients were thought to have one to three lesions (median 2) based on pre-GK imaging, which was predominantly MRI. These patients were discovered to have additional lesions at the time of the planning scan. On the day of treatment, patients harbored a median five lesions (range 4–12) with a median total tumor volume of 1.2 cc (range 0.11–15.7). At least one metastasis was located infratentorially in 27 cases (71%). A median dose of 21 Gy (range 15–21) was prescribed to the 50% isodose line to a median total treatment volume of 5.4 cc (range 0.97–24.5).

**Table 1:** Patient and disease characteristics

Characteristic	Study cohort (*n* = 38)
	Number of patients, (%)
Age at GK, years	
Median [range]	66 [30–85]
Gender	
Female	20 (53)
Male	18 (47)
Primary tumor	
NSCLC	17 (45)
Melanoma	14 (37)
Breast	3 (8)
Bladder	1 (3)
Ovarian	1 (3)
Renal cell	1 (3)
Mesothelioma	1 (3)
Extracranial disease	
None	2 (5)
Stable	5 (13)
Progressive	14 (37)
Newly diagnosed	17 (45)
RPA class^1^	
II	33 (87)
III	2 (5)
Unclassified	3 (8)
Primary to brain met, months	
Median [range]	4.2 [0–168]
Number of metastases	
4	12 (32)
5	10 (26)
6	6 (16)
7	2 (5)
8	4 (11)
9	1 (3)
10	0 (0)
11	1 (3)
12	2 (5)
Metastases location	
≥1 infratentorial	27 (71)
Total tumor volume, cc	
Median [range]	1.2 [0.1–15.7]
<3 cc	32 (84)
≥3 cc	6 (16)
Marginal dose, Gy	
Median [range]	21 [15–21]
Total treatment volume, cc	
Median [range]	5.4 [0.97–24.5]
<7 cc	24 (63)
≥7 cc	14 (37)

GK, Gamma Knife; NSCLC, non-small cell lung cancer; RPA, recursive partitioning analysis; met, metastases; cc, cubic centimeter; Gy, gray.

1 Five patients had performance status assigned retrospectively.

### Overall survival

**Figure 1:**
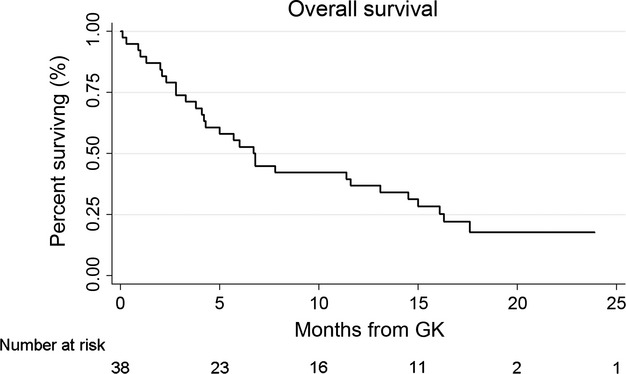
Overall survival from date of Gamma Knife (GK) stereotactic radiosurgery (SRS).

**Figure 2:**
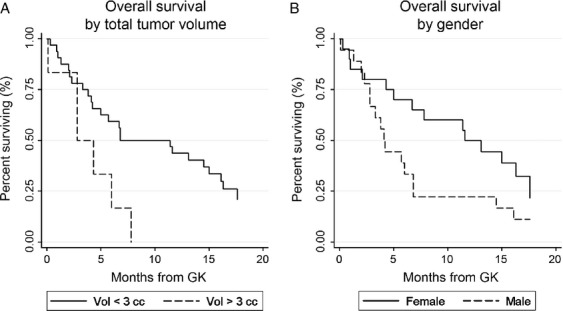
Overall survival by total tumor volume (A; *P* = 0.040, HR = 3.3, 95% CI 1.05–10.09) and gender (B; *P* = 0.079, HR = 1.9, 95% CI 0.93–4.08).

At the time of analysis, 30 patients (79%) had died. The median follow-up for the eight living patients was 17 months (range 12–29) and for the entire cohort was 6.8 months (range 0.1–29). Kaplan–Meier analysis revealed a median overall survival of 6.7 months from GK, with 74% alive at 3 months, 53% at 6 months, 42% at 9 months, and 36% at 12 months (Fig. 1). On univariate analysis, total tumor volume ≥3 cc (*P* = 0.02) was associated with worsened overall survival and male gender (*P* = 0.07) showed a trend suggesting association. Additional factors included in the multivariate model are listed in Table 2. On multivariate analysis, total tumor volume ≥3 cc was significantly associated with worsened survival (*P* = 0.040, HR = 3.3, 95% CI 1.05–10.09) and male gender showed a trend suggesting association (*P* = 0.079, HR = 1.9, 95% CI 0.93–4.08). Kaplan–Meier survival curves stratified by total tumor volume and gender are shown in Figure 2.

**Table 2:** Multivariate analyses

	*P*-value for individual outcome measures	
	
Characteristic	Overall survival	Distant failure
Male	0.079	0.043*
Age over 50 years	–	0.426
Melanoma primary	0.768	0.756
Lung primary	0.687	0.488
Five or more metastases	–	0.246
Nine or more metastases	0.112	–
Tumor volume ≥3 cc	0.040*	0.042*
Treatment volume >7 cc	–	0.142

cc, cubic centimeter.

* Statistically significant at *P* < 0.05 level.

### Intracranial failure

**Figure 3:**
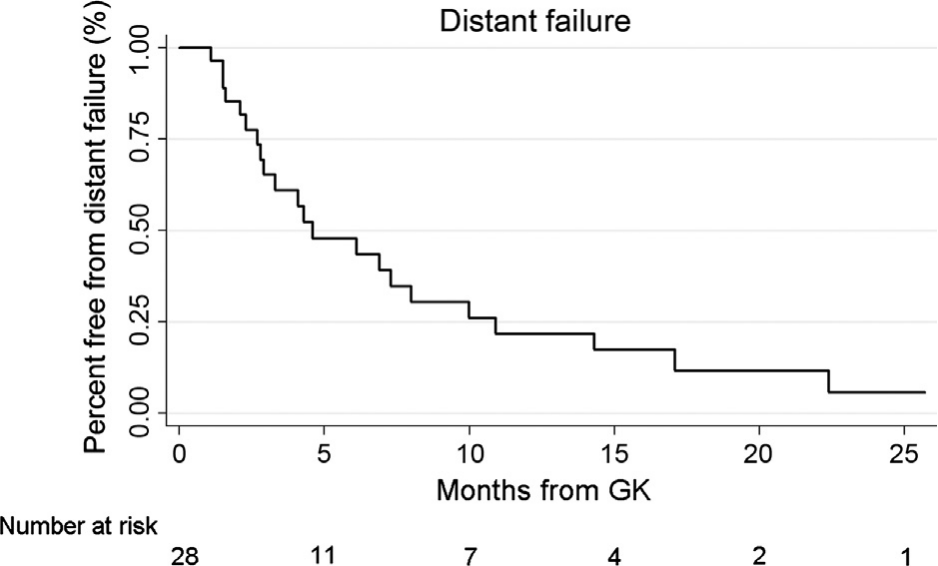
Distant tumor failure from date of Gamma Knife (GK) stereotactic radiosurgery (SRS).

**Figure 4:**
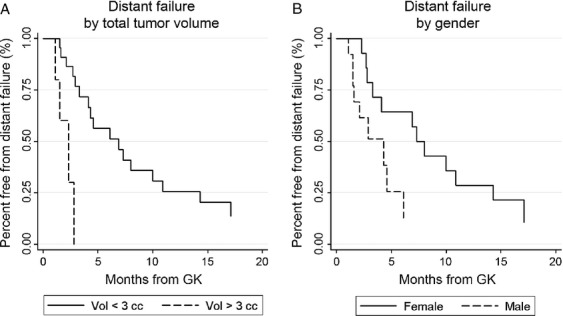
Distant tumor failure by total tumor volume (A; *P* = 0.042, HR = 6.1, 95% CI 1.07–35.40) and gender (B; *P* = 0.043, HR = 2.8, 95% CI 1.03–7.52).

Analysis of the 28 patients with follow-up imaging showed that distant failure occurred in 22 cases (79%) and local failure occurred in one case (4%). Kaplan–Meier analysis revealed a median time to distant failure of 4.6 months from GK, with 65% free from distant failure at 3 months, 48% at 6 months, 30% at 9 months, and 22% at 12 months (Fig. 3). On univariate analysis, factors associated with distant failure were total tumor volume ≥3 cc (*P* = 0.0008), melanoma primary (*P* = 0.02), and treatment volume ≥7 cc (*P* = 0.05). Lung primary (*P* = 0.06) and male gender (*P* = 0.09) showed a trend suggesting association. Additional factors included in the multivariate model are listed in Table 2. On multivariate analysis, both total tumor volume ≥3 cc (*P* = 0.042, HR = 6.1, 95% CI 1.07–35.40) and male gender (*P* = 0.043, HR = 2.8, 95% CI 1.03–7.52) were significantly associated with distant failure. Kaplan–Meier curves of distant failure stratified by total tumor volume and gender are shown in Figure 4.

### Salvage therapies

**Figure 5:**
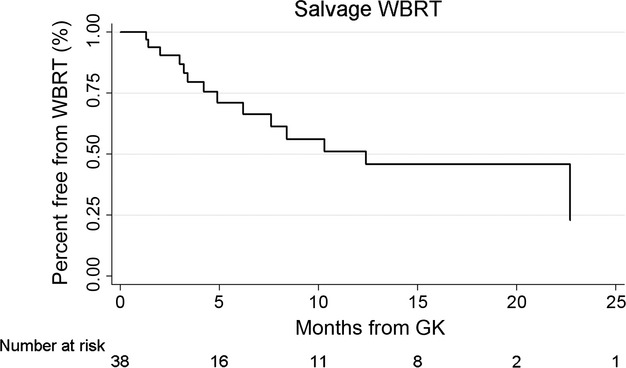
Salvage whole-brain radiation therapy from date of Gamma Knife (GK) stereotactic radiosurgery (SRS).

Nineteen patients (50%) underwent some form of salvage therapy during their course of care. Salvage WBRT was given to 14 patients (37%). Kaplan–Meier analysis of all cases revealed a median time to WBRT of 10.3 months, with 87% free from WBRT at 3 months, 71% at 6 months, and 56% at 9 months (Fig. 5). Seven patients (18%) received repeat GK at a median 5.5 months from initial GK (range 2.5–12.2). One patient had two repeat GK sessions. One patient underwent Cyberknife in addition to two surgeries for symptomatic hemorrhage of treated metastases.

### Adverse events

One patient had symptomatic radionecrosis of two treated lesions and required steroid treatment. One patient developed radiation injury to the cerebellum resulting in gait ataxia. A single patient had seizures within a week of GK and was treated with steroids and anticonvulsants. Radiologic evidence of leptomeningeal carcinomatosis developed in five patients (13%).

## Discussion

Prospective, randomized trials have explored SRS for a limited number of brain metastases, with enrollment restricted to patients harboring one to three lesions in all but two studies 1–6. Kondziolka et al. allowed up to four lesions but included only four such patients 6. Aoyama examined cases with one to four lesions but the number of patients with >3 metastases was not reported 5. In total, these studies suggest SRS added to WBRT confers a survival benefit in cases of a single lesion and improves local and distant control for either single or multiple lesions.

Data are more limited on the role of SRS for patients with four or more brain metastases, and existing evidence is retrospective. WBRT remains the standard of care in such situations 7,8. Potential advantages of sole therapy with SRS are twofold: single-session treatment allows faster access to systemic chemotherapy and SRS may avoid the neurocognitive concerns associated with WBRT 3. Radiosurgery has recently emerged as a viable treatment strategy for patients with multiple lesions, and this shift is captured in two sets of expert guidelines. While the American College of Radiology's 2009 guidelines cite a lack of evidence for SRS alone and rate it as the least appropriate management of multiple metastases 7, the American Society for Radiation Oncology's 2012 guidelines reference the growing literature on this strategy, acknowledge that the number or volume of brain metastases suitable for radiosurgery is not clear, and state that proceeding with SRS alone in the case of multiple metastases can be a reasonable treatment strategy 8.

Existing studies on SRS to ≥4 metastases are confounded by significant proportions of patients treated with prior WBRT or concurrent WBRT along with SRS (e.g. 60% 10, 69% 12, 77% 16, and 84% 11). In contrast, our study examines this treatment strategy in a population receiving SRS alone without a prior history of radiation or surgery.

Previous reports have consistently demonstrated that increasing number of lesions beyond four is not significantly associated with worsened survival 10,11,14–18, but other prognostic factors remain in dispute. In particular, published results disagree about the impact of tumor volume. Bhatnagar et al. reported total tumor volume as the strongest prognostic factor for survival in patients with ≥4 lesions and suggested volume as the better selection criterion for SRS than number of lesions 11. This echoed earlier findings by Jawahar et al. 9. In direct contrast, both DiLuna et al. and Nam et al. explicitly report no correlation between survival and total volume of metastases 10,12.

In this retrospective study, we examined GK as sole initial therapy for multiple metastases in a cohort of patients with no prior history of intracranial radiation or surgery. Our median survival of 6.7 months after GK alone compares favorably to results with WBRT alone in patients with ≥4 lesions, as reported by Nieder et al. (3.6 and 4.2 months for RPA Class II and III, respectively) 19. Our multivariate analyses support the view that instead of number of metastases, it is the total volume of lesions that predicts worsened survival, and we report a cutoff value of 3 cc. Male gender was significantly associated with distant failure and showed a trend suggesting association with overall survival, even when controlling for primary tumor type. These results support the findings of Serizawa et al. that male gender is an independent risk factor in overall survival 14,18.

Our study revealed good local control, but most patients (79%) did develop distant brain disease. These recurrences were managed with further GK, surgery, or salvage WBRT. A strategy of upfront SRS avoided WBRT in 67% of patients. Those who ultimately underwent salvage WBRT had it delayed for multiple months from the date of GK.

There are several limitations to this study, including its retrospective nature and small size. Selection bias may have influenced which patients were referred for GK over WBRT, and we were unable to assign RPA class in three cases due to insufficient information about performance status. An important limitation was our lack of data on neurocognitive outcomes. To this end, the North American Gamma Knife Consortium is opening a randomized trial of WBRT versus SRS in patients with five or more brain metastases. The primary aim is to evaluate changes in neurocognitive outcome between the two groups. Our study adds further support to the existing evidence that SRS for multiple lesions is a reasonable strategy, and it should encourage clinicians to enroll their patients onto the upcoming randomized trial.

## Conclusion

GK radiosurgery as sole initial treatment for four or more simultaneous metastases spares some patients WBRT and delays it for others. Increased total tumor volume (≥3 cc) is significantly associated with worsened overall survival.
